# Quantifying Joule Heating and Mass Transport in Metal Nanowires during Controlled Electromigration

**DOI:** 10.3390/ma12020310

**Published:** 2019-01-19

**Authors:** Mamiko Yagi, Jun-ichi Shirakashi

**Affiliations:** 1 Division of Electrical and Electronic Engineering, Department of Engineering for Future Innovation, Ichinoseki College (Ichinoseki KOSEN), Ichinoseki, Iwate 021-8511, Japan; m-yagi@ichinoseki.ac.jp; 2 Department of Electrical and Electronic Engineering, Tokyo University of Agriculture and Technology, Koganei, Tokyo 184-8588, Japan

**Keywords:** atomic force microscopy, electromigration, nanowire, Joule heating, nanogap

## Abstract

The nanoscale heat dissipation (Joule heating) and mass transport during electromigration (EM) have attracted considerable attention in recent years. Here, the EM-driven movement of voids in gold (Au) nanowires of different shapes (width range: 50–300 nm) was directly observed by performing atomic force microscopy. Using the data, we determined the average mass transport rate to be 10^5^ to 10^6^ atoms/s. We investigated the heat dissipation in L-shaped, straight-shaped, and bowtie-shaped nanowires. The maximum Joule heating power of the straight-shaped nanowires was three times that of the bowtie-shaped nanowires, indicating that EM in the latter can be triggered by lower power. Based on the power dissipated by the nanowires, the local temperature during EM was estimated. Both the local temperature and junction voltage of the bowtie-shaped nanowires increased with the decrease in the Joule heating power and current, while the current density remained in the order of 10^8^ A/cm^2^. The straight-shaped nanowires exhibited the same tendency. The local temperature at each feedback point could be simply estimated using the diffusive heat transport relationship. These results suggest that the EM-driven mass transport can be controlled at temperatures much lower than the melting point of Au.

## 1. Introduction

The electromigration (EM) phenomenon is of high technological importance for the semiconductor industry, as it is the main cause of failure in integrated circuits [1,2]. The dissipative heating at the interconnect is considered the dominant driving force behind EM, with the cumulative momentum transfer from conduction electrons to thermally activated ions in a conductor. These negative effects can be, if properly tuned, used to our advantage to prepare nanogap electrodes made of metal nanowires for fabricating quantum tunneling devices [3]. This opens the possibility for an alternative method based on only the current passing through a metal nanowire without requiring conventional lithography techniques. However, the EM-generated nanogap procedure with a simple voltage ramp often results in poorly tuned tunnel resistance, which can ultimately destroy the nanowires [3]. To prevent thermal runaway and enable temperature tuning in nanowires, feedback-controlled electromigration (FCE) methods based on resistance/conductance monitoring have been developed [4,5,6,7,8,9]. Although resistance/conductance measurements reflect the structural changes in the nanowires during EM [9], they do not give enough insight into the mechanism of nanogap formation. A real-time imaging of the morphological changes during EM is essential. In our previous studies, the mass transport in electromigrated Au nanowires was investigated using an in situ atomic force microscopy (AFM) technique in ambient air [10]. The established in situ AFM technique helped to better understand the relationship between the wire geometry, current density, and heat dissipation (Joule heating) during FCE. To fabricate functional nanoscale devices such as single-electron transistors [11,12] and single-molecule transistors [13], it is important to quantitatively study the mass transport and local temperature in the nanowires, and better understand the void movements (moving atoms) during EM. Here, we focus on the changes in the morphology of voids in Au nanowires induced by EM, and the local temperature during FCE in Au nanowires observed by performing AFM topography using the diffusive heat transport relationship. Moreover, the effect of the heat dissipation near the nanoconstriction on the nanowire shape is investigated.

## 2. Experimental Method

**Figure 1:**
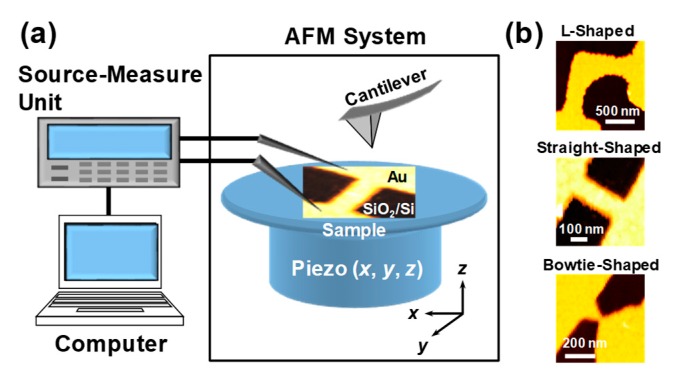
(**a**) Schematic of the experimental setup for in situ atomic force microscopy (AFM) imaging; (**b**) AFM images of Au nanowires of different shapes.

Figure 1a shows the schematic of the experimental setup. The Au nanowires used in this study were defined using electron-beam lithography, evaporation, and lift-off technique on Si/SiO_2_ substrates. L-shaped, straight-shaped, and bowtie-shaped Au nanowires were fabricated as shown in Figure 1b. The measurements reported in this study were performed in air at room temperature and ambient atmospheric pressure. The morphological modification of the nanowires during FCE was quantitatively evaluated using SPA400/SPI4000 (SII Nanotechnology, Inc., Tokyo, Japan). The AFM measurements were carried out in the contact mode using commercially available silicon nitride cantilevers (Olympus Co., Tokyo, Japan) with a spring constant of 0.02 N/m. The frequency of the scan comprising 256(*x*) × 128(*y*) data points was in the range of 5–10 Hz, and the images were obtained in approximately 15–30 s. Each nanowire was placed on a specially designed AFM holder, via which an electric current could be applied through the sample during AFM observation. Here, we focus on the significant structural changes in the nanowires during FCE at the cathode side, due to negligible Joule heating and prominent EM. We captured real-time AFM images of the nanowires from 200 and 500 nm^2^ topographic scans in the vicinity of the cathode end. It is now generally accepted that the junction resistance *R*_J_ is significantly lower than the lead resistance *R*_L_. In this case, *R*_J_ was approximately in the range of 1–4 Ω, and the overall initial resistance *R* (=*R*_J_ + *R*_L_) of the samples was approximately in the range of 33–69 Ω.

A feedback control scheme, which is reported elsewhere [4], was used to apply the electrical stresses to the nanowires. The current–voltage (*I*–*V*) characteristics of the nanowires were recorded using a computer-controlled source-measure unit (Keithley 2400, Solon, OH, USA). The voltage *V* applied to the nanowires was automatically increased in constant voltage steps *V*_STEP_, and the conductance *G* was monitored. When the differential conductance Δ*I*/Δ*V* reaches the threshold differential conductance *G*_TH_, the program reduces the voltage by an amount equal to the feedback (FB) voltage *V*_FB_ to avoid a catastrophic failure. These voltage reduction events are further referred to as FB points. Subsequently, the voltage ramp was reinitiated continuously.

## 3. Results and Discussion

### 3.1. In Situ AFM Imaging of Au Nanowires during FCE

**Figure 2:**
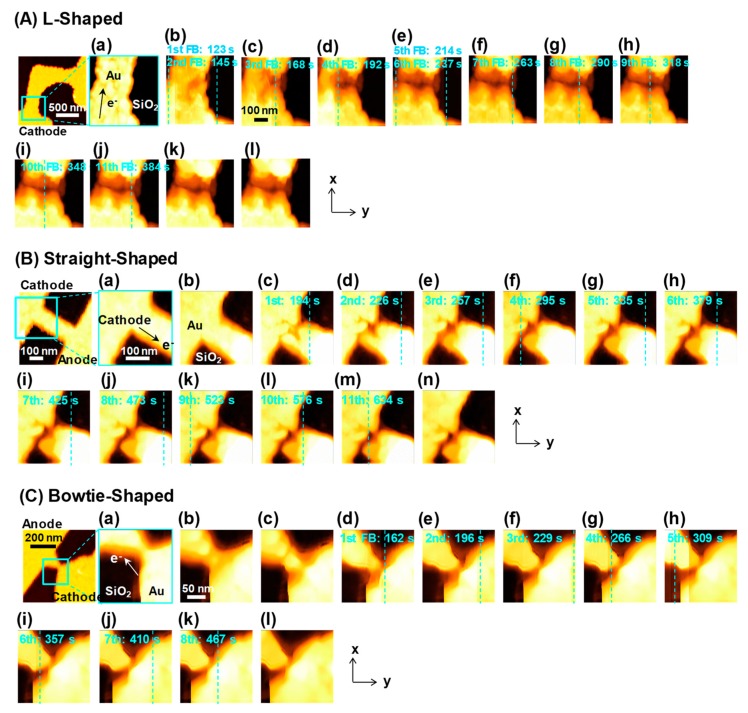
(**A**) Atomic force microscopy (AFM) images of the L-shaped nanowires captured with a scanning window of 500 × 500 nm^2^. The images were captured during the following time intervals: (**a**) *t* = 92–122 s, (**b**) *t* = 122–153 s, (**c**) *t* = 153–183 s, (**d**) *t* = 183–213 s, (**e**) *t* = 213–244 s, (**f**) *t* = 244–274 s, (**g**) *t* = 274–305 s, (**h**) *t* = 305–335 s, (**i**) *t* = 335–366 s, (**j**) *t* = 366–396 s, (**k**) *t* = 396–427 s, and (**l**) *t* = 457–488 s. The light-blue dashed lines in (**b**–**j**) indicate each scan line from the 1st to the 11th corresponding feedback (FB) point. The time indicated at the top of each image is the time corresponding to each FB point. (**B**) AFM images of the straight-shaped nanowires captured with a scanning window of 300 × 300 nm^2^. The images were captured during the following time intervals: (**a**) *t* = 0–15 s, (**b**) *t* = 168–184 s, (**c**) *t* = 184–199 s, (**d**) *t* = 214–230 s, (**e**) *t* = 245–260 s, (**f**) *t* = 291–306 s, (**g**) *t* = 322–337 s, (**h**) *t* = 368–383 s, (**i**) *t* = 414–429 s, (**j**) *t* = 460–475 s, (**k**) *t* = 521–536 s, (**l**) *t* = 567–582 s, (**m**) *t* = 628–644 s, and (**n**) *t* = 705–720 s. The light-blue dashed lines in (**c**–**m**) indicate each scan line from the 1st to the 11th corresponding FB point. The time indicated at the top of each image is the time corresponding to each FB point. (**C**) AFM images of the bowtie-shaped nanowires captured with the scanning window of 200 × 200 nm^2^. The images were captured during the following time intervals: (**a**) *t* = 0–15 s, (**b**) *t* = 122–138 s, (**c**) *t* = 138–153 s, (**d**) *t* = 153–168 s, (**e**) *t* = 184–199 s, (**f**) *t* = 214–230 s, (**g**) *t* = 261–276 s, (**h**) *t* = 307–322 s, (**i**) *t* = 353–368 s, (**j**) *t* = 399–414 s, (**k**) *t* = 460–475 s, (**l**) *t* = 521–537 s. The light-blue dashed lines in (**d**–**k**) indicate each scan line from the 1st to the 8th corresponding FB point. The time indicated at the top of each image corresponds to the time of each FB point.

Figure 2(Aa–l) shows the AFM images of the L-shaped Au nanowires of length 1.5 μm, captured during 92–488 s. The blue dashed lines in Figure 2(Ab–j) indicate the scan positions at each FB point, and the time corresponding to each FB point is indicated in each of the images. The electron flow is from down (cathode) to up (anode). The results show that the structural changes in the nanoconstriction were gradual, and from the 1st to the 11th FB point.

**Figure 3:**
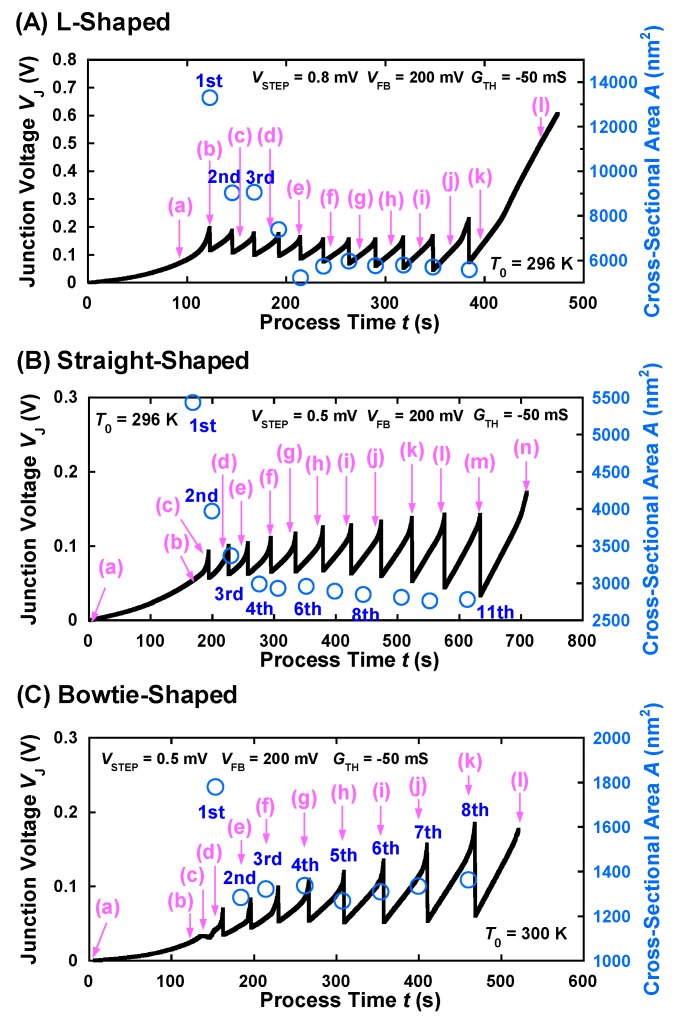
Time evolution of the *V*_J_–*A* characteristics of the (**A**) L-shaped, (**B**) straight-shaped, and (**C**) bowtie-shaped nanowires during feedback-controlled electromigration (FCE).

Similar results were obtained for the straight-shaped and bowtie-shaped nanowires of length 300 nm, as shown in Figure 2(Ba–n) and Figure 2(Ca–l), respectively. In Figure 2B, the electrons flow from upper left (cathode) to lower right (anode), whereas in Figure 2C, they move from lower right (cathode) to upper left (anode). Despite different scan directions, in contrast to a direction of electron flow, the EM-induced mass transport in Au nanowires of different shapes was observed in the vicinity of the cathode end, indicating that void movements (moving atoms) during EM did not suffer from the scan of the cantilever. Figure 3A shows the time evolutions of the junction voltage *V*_J_ and the cross-sectional area *A* of the L-shaped nanowires during the FCE process for *V*_STEP_ = 0.8 mV, *V*_FB_ = 200 mV, and *G*_TH_ = −50 mS. The starting points of the scans, shown in Figure 2(Aa–l), are indicated using pink arrows, as shown in Figure 3A. To calculate *A* from the AFM images of the nanowire, the SiO_2_ surface observed in the images was used. The cross sections perpendicular to the direction of electron flow were taken near the nanoconstriction. Each open circle represents the *A* value of the image including the automatic decrease in the voltage by *V*_FB_. There was a decrease in *A* from 13,310 nm^2^ (1st FB point) to 5597 nm^2^ (11th FB point) during the voltage feedback process, indicating that the FCE process thinned down the nanowire without any thermal runaway or melting.

**Figure 4:**
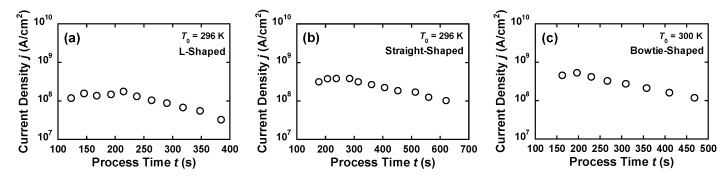
Current densities of the (**a**) L-shaped, (**b**) straight-shaped, and (**c**) bowtie-shaped nanowires as a function of the FCE process time.

Similarly, Figure 3B,C shows the time evolutions of *V*_J_ and *A* of the straight-shaped and bowtie-shaped nanowires, respectively, during the FCE process for *V*_STEP_ = 0.5 mV, *V*_FB_ = 200 mV, and *G*_TH_ = −50 mS. The pink arrows, indicated in Figure 3B,C, correspond to the starting points of the scans shown in Figure 2(Ba–n) and Figure 2(Ca–l), respectively. As shown in Figure 3B, *A* of the straight-shaped nanowires reduced from 5437 nm^2^ (1st FB point) to 2996 nm^2^ (4th FB point) and gradually decreased to 2766 nm^2^ (11th FB point). Figure 3C shows the gradual decrease in *A* of the bowtie-shaped nanowires, from 1781 to 1271 nm^2^. The *V*_J_–*A* characteristics confirm that the rapid EM of the Au atoms was suppressed under the feedback algorithm. Figure 4a–c shows the current density *j* of the L-shaped, straight-shaped, and bowtie-shaped nanowires as a function of the FCE process time. Notably, despite the different shapes of the nanowires, the value *j* (obtained by dividing *I* by *A*) remains constant, in the order of 10^8^ A/cm^2^ during the FCE process. The roughly estimated *j* value is reasonably consistent with previous reports on the failure of the current density of Au nanowires [6,7,14,15].

### 3.2. Mass Transport in Electromigrated Au Nanowires

The quantitative mass transport used to characterize the EM process in the nanowires can be obtained from the measured nanowire topographies. We defined the SiO_2_ surface observed in the images as the reference plane. The Au mass transport was calculated by summing the differential volumes in the vicinity of the cathode side, before and after the FB point during the FCE process, and then multiplying the result with the Au density [10]. The change in the volume between the images can be expressed as follows:

(1) $$\Delta U_{\mathrm{ab}}\left({\mathrm{nm}}^{3}\right)=\underset{x,y}{\sum }\left(f_{\mathrm{axy}}\Delta S_{a}-f_{\mathrm{bxy}}\Delta S_{b}\right)$$

where *a* and *b* are the image indices, *x* and *y* are the positions in the image, Δ*S*_a_ (=Δ*S*_b_) (nm^2^) is the total area of the image (nm^2^)/(256 × 128 data points), and *f*_axy_ and *f*_bxy_ (nm) are the heights of the points with respect to the reference plane. The Au mass Δ*M*_ab_ transported from the cathode can be expressed as follows.

(2) $$\Delta M_{\mathrm{ab}}\left(\mathrm{atoms}\right)=\Delta U_{\mathrm{ab}}{(\mathrm{nm}}^{3}{)\times d\;(\mathrm{atoms}/\mathrm{nm}}^{3})$$

where *d* is the atom density (=58.9 atoms/nm^3^) [16], assuming that the Au nanowire has an FCC structure. We determined the rate of mass transport Δ*R*_ab_ in time intervals of 30 s between two AFM images. Finally, the average values Δ*M*_AVE_ and Δ*R*_AVE_ of Δ*M*_ab_ and Δ*R*_ab_ were obtained, as listed in Table 1. The average values (Δ*R*_AVE_) of the mass transport rate were in reasonable agreement with the values reported for real-time TEM imaging of EM-induced nanogap formation, that is, 10^6^ atoms/s (=10^5^ atoms/50 ms) [17]. The straight-shaped and bowtie-shaped nanowires had relatively lower Δ*R*_AVE_ values than the L-shaped nanowire. The relatively low Δ*R*_AVE_ could be a result of the smaller cross-sectional area *A* and lower constant voltage step *V*_STEP_. To investigate the influence of the temperature (due to the Joule heating) required to control the lower mass transport, we first used the constant power model for the three nanowires.

### 3.3. Nanoscale Heat Dissipation in Electromigrated Au Nanowires

**Figure 5:**
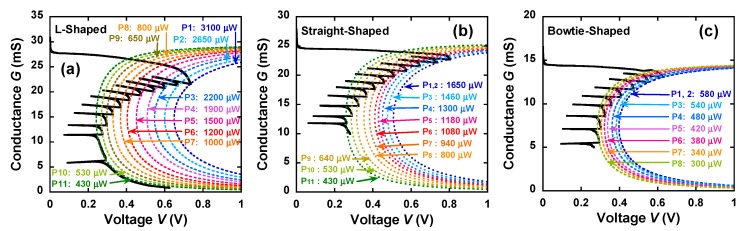
*G* as a function of the total voltage *V* for the (**a**) L-shaped, (**b**) straight-shaped, and (**c**) bowtie-shaped nanowires during FCE. The dashed lines indicate the fitting results of the Joule heating model.

**Figure 6:**
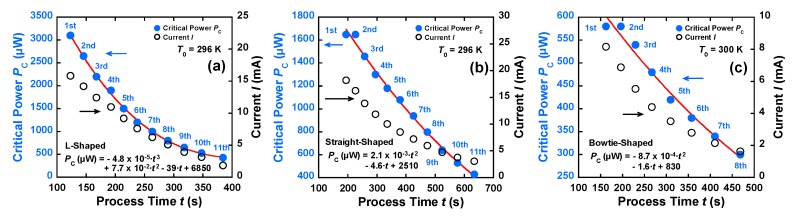
Time evolution of the *P*_C_–*I* characteristics of the (**a**) L-shaped, (**b**) straight-shaped, and (**c**) bowtie-shaped nanowires as a function of the FCE process time. The red lines indicate the fitting result of *P*_C_ (μW): (**a**) −4.8 × 10^−5^·*t*^3^ + 7.7 × 10^−2^·*t*^2^ − 39·*t* + 6850 (*R* = 0.999), (**b**) 2.1 × 10^−3^·*t*^2^ − 4.6·*t* + 2510 (*R* = 0.997), and (**c**) −8.7 × 10^−4^·*t*^2^ − 1.6·*t* + 830 (*R* = 0.991).

The overall curve with some FB points can be modeled using the following equation [4,6]:

(3) $${V=V}_{J}{+V}_{L}=\frac{P_{J}}{I}{+R}_{L}I$$

where *V* is the total voltage applied to the device, *V*_J_ and *V*_L_ [V] are the voltages across the constriction and leads, respectively, *P*_J_ (μW) is the power dissipated at the nanoconstriction, and *I* (mA) is the current. The curves shown in Figure 5a–c represent the fit of Equation (3) to the *G*–*V* characteristics of the nanowires during the FCE process. *P*_J_ at each FB point was defined as the critical power *P*_C_. *P*_C_ was fitted to the model and was found to decrease from 1650 μW (at the first and second FB points) to 430 μW (at the 11th FB point) for the straight-shaped nanowires, shown in Figure 5b, and from 580 μW (at the first and second FB points) to 300 μW (at the 8th FB point) for the bowtie-shaped nanowires, shown in Figure 5c. For the L-shaped nanowires shown in Figure 5a *P*_C_ ranged from 3100 μW (at the first FB point) to 430 μW (at the 11th FB point). Figure 6a–c shows the *P*_C_ and *I* values as a function of the FCE process time. The red lines in Figure 6a–c indicate the fitting result of *P*_C_. *P*_C_ reduced with the decrease in *I*, which is consistent with previous reports [9,18]. The smaller the nanoconstriction, the lower the critical power consumed during the FCE process. The tapered region in the bowtie-shaped nanowires may allow easy flow of Joule heating generated in the nanoconstriction. Thinner and narrower wires—particularly ones made of Au—are known to generate lower heat when the electron–phonon scattering length is on the order of 170 nm [15]. Thus, the mass transport can be precisely tuned using a lower Joule heating power. For metallic constrictions in the diffusive regime, the scale of the voltage due to the constriction resistance is associated with the temperature scale [6,19].

(4) $$T^{2}=T_{0}{}^{2}+\frac{V_{J}{}^{2}}{4L}$$

where *L* (V^2^/K^2^) (=(*π*^2^/3)(*k*/*e*)^2^) is the Lorenz number, and *T* and *T*_0_ (K) are the temperatures of the constriction and its environment, respectively. This relationship can be theoretically deduced, assuming that the electrical conductivity is related to the thermal conductivity (Wiedemann–Franz law) [6,19]. In this study, at the onset of EM, the local temperature *T* at the constriction was defined as the critical local temperature *T*_C_, and *V*_J_ was defined as the critical junction voltage *V*_C_. Thus, at each FB point, *V*_C_ could be calculated with respect to the time evolution by dividing *P*_C_ by *I*. When *T* at the constriction is equal to *T*_C_, *T*_C_ at each FB point can be simply estimated using the diffusive heat transport relationship as follows [6,19]:

(5) $$T_{C}=\sqrt{T_{0}{}^{2}+\frac{V_{C}{}^{2}}{4L}}=\sqrt{T_{0}{}^{2}+\frac{P_{C}{}^{2}}{4I^{2}L}}$$

**Figure 7:**
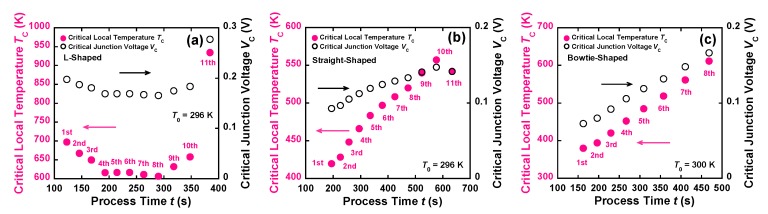
Time evolution of the *T*_C_–*V*_C_ characteristics of the (**a**) L-shaped, (**b**) straight-shaped, and (**c**) bowtie-shaped nanowires as a function of the FCE process time.

According to Equation (5), *T*_C_ depends on *V*_C_. Figure 7a–c shows the time evolutions of *T*_C_ and *V*_C_ under the feedback algorithm. Table 2 presents the relationship between *T*_C_, *V*_C_, *P*_C_, and *I*, demonstrating the range of electrical and thermal properties at the FB points of the three nanowires. The *T*_C_ value of the bowtie-shaped nanowires increased from 379 to 612 K at *T*_0_ = 300 K with the increase in *V*_C_ from 0.073 to 0.17 V. Both *T*_C_ and *V*_C_ linearly varied with the decrease in *P*_C_ and *I*. This tendency was quite similar to that exhibited by the straight-shaped nanowires. The estimated values were in reasonable agreement with previous reports (325–660 K) [7,8,9,14,20,21]. In the case of the L-shaped nanowires, although *T*_C_ decreased from 697 K (at the 1st FB point) to 611 K (at the 7th FB point), *T*_C_ increased from 607 K (at the 8th FB point) to 935 K (at the 11th FB point) in the same manner as that observed for the straight-shaped and bowtie-shaped nanowires. This implies that the longer and wider L-shaped nanowires consumed more power to approximately the 7th FB point, whereas the behavior of the narrower L-shaped nanowires was similar to the behavior of the straight-shaped and bowtie-shaped nanowires after approximately the 7th FB point. The higher value of *T*_C_ at the 11th FB point could have been due to the accumulation of Joule heating for a long time between the 10th and 11th FB points. As shown in Figure 2(Al), at the end of the FCE process, there was no abrupt failure of the nanoconstriction due to melting, because the local temperature was significantly lower than the melting point of gold (~1337 K) [22]. The *T*_C_ values at each FB point could be roughly interpreted using the diffusive heat transport relationship, and the value of *T*_C_ at the 1st FB point was related to the wire size. These results confirm the EM-induced mass transport phenomenon in Au nanowires from room temperature to temperatures significantly lower than the melting point of Au, with *T*_C_ increasing in a controlled manner independent of the nanowire shape.

## 4. Conclusions

We studied the mass transport and local Joule heating in Au nanowires during EM in real-time by performing AFM in air at room temperature and ambient atmospheric pressure. The applied voltage and current measurements and cross-sectional area of the nanoconstriction obtained during FCE indicated that the EM-induced mass transport proceeded in the order of 10^8^ A/cm^2^ in a gradual manner under the application of a voltage feedback algorithm. The estimated average mass transport was in the order of 10^5^ to 10^6^ atoms/s; the straight-shaped and bowtie-shaped nanowires gave lower values. Furthermore, based on the power dissipated by the nanowires, we found that the local temperature in the nanoconstriction during FCE ranged from room temperature to temperatures lower than the melting point of Au. The results suggest that the accumulation and movement of the voids were induced by EM without any melting of the large parts of the nanowires due to Joule heating. Moreover, the control of the mass transport during EM hardly depended on the shape of the nanowires. The results of this study are expected to provide more insight into the matter of fluxes during EM. The technological implications of this work are that if bowtie-shaped nanowires are used to prepare nanogap electrodes, then in order to control and observe lower mass transport, they must be as narrow as possible, while remaining as short as possible.

## Figures and Tables

**Table 1:** FB parameters, average mass transport values, and average rates of mass transport for each Au nanowire.

Nanowire	*V*_STEP_ (mV)	*V*_FB_ (mV)	*G*_TH_ (mS)	Δ*M*_AVE_ (atoms)	Δ*R*_AVE_ (atoms/s)
L-Shaped	0.8	200	−50	2.3 × 10^7^	7.6 × 10^5^
Straight-Shaped	0.5	200	−50	2.3 × 10^6^	7.7 × 10^4^
Bowtie-Shaped	0.5	200	−50	2.0 × 10^6^	6.6 × 10^4^

**Table 2:** Critical parameter ranges for the L-shaped, straight-shaped, and bowtie-shaped nanowires.

Nanowire	*I* (mA)	*P*_C_ (µW)	*V*_C_ (V)	*T*_C_ (K)
L-Shaped	1.84–15.9	430–3100	0.17–0.28	607–935
Straight-Shaped	3.08–18.2	430–1650	0.093–0.15	420–557
Bowtie-Shaped	1.63–8.16	300–580	0.073–0.17	379–612
